# Lewy body dementia: exploring biomarkers and pathogenic interactions of amyloid β, tau, and α-synuclein

**DOI:** 10.1186/s13024-025-00879-0

**Published:** 2025-08-12

**Authors:** Jingfeng Liang, Rongzhen Li, Garry Wong, Xiaobing Huang

**Affiliations:** 1 College of Pharmacy, Shenzhen Technology University, Shenzhen, 518000 China; 2 Department of Neurology, Baiyun District People’s Hospital of Guangzhou, Guangzhou, 510000 China; 3 Department of Global Public Health and Medicinal Administration, Faculty of Health Sciences, University of Macau, Macau S.A.R, 999078 China

**Keywords:** Lewy body dementia, Dementia with Lewy bodies, Parkinson’s disease dementia, Amyloid β, Tau, Α-synuclein

## Abstract

Lewy body dementia (LBD) is a neurodegenerative disorder characterized by a combination of progressive dementia and spontaneous parkinsonian symptoms. As the second most prevalent form of neurodegenerative dementia after Alzheimer’s disease (AD), LBD necessitates a deeper understanding of its pathogenesis to enable the development of targeted therapeutic interventions. While numerous reviews focus on documenting the clinical manifestations and therapeutic modalities for LBD, animal models provide valuable insights into the underlying mechanisms and potential therapeutic strategies. In this review, we systematically analyze the hallmarks of LBD pathogenesis, genetic risk factors, clinical features, and treatment strategies. Importantly, we emphasize and critically evaluate the pivotal role of animal models in LBD research in advancing our understanding of this disorder, offering a comprehensive framework to elucidate the interactions among misfolded proteins and their role in LBD pathogenesis. Our review proposes new directions for LBD therapeutic management and facilitates the development of innovative pharmacological interventions.

## Background

Dementia constitutes a multifaceted clinical syndrome characterized by progressive deterioration of cognitive functions, encompassing memory, language, intellectual processes, and problem-solving abilities, which substantially interferes with the performance of routine daily activities. The most prevalent forms of neurodegenerative dementia include Alzheimer’s disease (AD), accounting for 60–80% of cases, followed by Lewy body dementia (LBD), at 10–15%, Frontotemporal dementia (FTD) at 5–10%, and Vascular dementia (VaD) at 5–10% [1, 2]. AD receives significant clinical attention due to its high prevalence. However, LBD, recognized as a distinct entity since 1984, is less comprehensively characterized compared to AD and Parkinson’s disease (PD) [3].

LBD is a progressive neurodegenerative disorder defined by the co-occurrence of dementia and spontaneous parkinsonian features, encompassing two clinical subtypes: Dementia with Lewy bodies (DLB) and Parkinson’s disease dementia (PDD) [4]. As the second most prevalent type of neurodegenerative dementia, the annual incidence of DLB was reported as 3.8% of new dementia cases and 0.87 cases per 1000 person-years [5]. Its prevalence increases with age, affecting 0.3–24.4% of elderly individuals with dementia [6]. Major clinical manifestations include cognitive fluctuations, visual hallucinations, motor parkinsonism, and rapid eye movement (REM) sleep behavior disorder (RBD) [7]. Accurate differentiation between PDD, DLB, AD, and PD, is critical for the effective diagnosis and management of LBD.

## Differentiation between AD, PD, and LBD

### PDD vs. DLB

LBD, encompassing DLB and PDD; clinical differentiation between these two conditions is facilitated by the application of symptom onset, progression, and diagnostic criteria [7–9]. In DLB, dementia is the core feature, with parkinsonian motor symptoms emerging concurrently or within one year of the onset of cognitive decline, although it has been shown that 25%-30% of DLB patients never develop any parkinsonian symptoms [10, 11]. Conversely, in PDD, parkinsonian motor features must predate the development of dementia by a period of one year or longer [7–9]. Epidemiological studies indicate that approximately 10% of PD patients will subsequently develop dementia annually, with dementia prevalence reaching 74%-83% among 20-year PD survivors [12, 13]. The late-stage clinical manifestations and management strategies for both DLB and PDD are comparable, as they both present with a pair of cognitive impairment and parkinsonian features [14–16]. While DLB and PDD are marked by clinical heterogeneity, not all patients develop motor changes or exhibit all key clinical features. Therefore, this variability underscores the necessity of comprehensive assessment and individualized diagnosis for patients with LBD.

Subtle distinctions exist between DLB and PDD. DLB cases have been observed to exhibit higher levels of amyloid β (Aβ) and α-synuclein (α-syn) in the hippocampus, temporal, and parietal cortex when compared to PDD [17]. Besides, cortical α-syn pathology stands as the primary substrate candidate for PDD, whereas Aβ plays a more pronounced role in DLB [18]. Additionally, neurodegeneration in the ventrolateral substantia nigra is a feature of both PD and PDD, whereas it is less prevalent in DLB, which may contribute to the clinical observation of parkinsonism predating dementia in PDD [19].

Despite these pathological divergences, DLB and PDD share overlapping clinical presentations, with patients displaying cognitive decline, attentional fluctuations, visual hallucinations, and REM sleep disorder [7, 17]. A longitudinal Swedish cohort study of 177 patients diagnosed with DLB or PDD between 1997 and 2014 revealed a mortality rate that was over three times higher than that of the general population, with median survival of only 4.1 years for 81% of patients, while no significant survival difference emerged between DLB and PDD subgroups [20]. Given these clinical and pathological similarities, PDD and DLB may be more appropriately conceptualized as a spectrum under the encompassing term of LBD, rather than as two distinct entities.

### AD vs. LBD

Differentiating LBD from AD poses significant diagnostic challenges in clinical practice. AD is typically characterized by a gradual decline in memory, language, executive, and visuospatial functions, with advanced stages often presenting behavioral disinhibition and loss of fundamental daily living activities [21]. In contrast, LBD presentations often include emotional fluctuations, behavioral inconsistencies, variable attention, or incoherent speech [7]. Differentiating LBD from AD, which can also present with a dementia pattern, can be challenging. Some studies indicated that LBD patients may exhibit more severe impairments in visuospatial abilities, attention, and executive function [22–24]. A key feature of LBD is the onset of complex visual hallucinations, which suggests the presence of Lewy bodies disease [7]. About 61.8%-76% of LBD patients experience these hallucinations, making them a frequent and potentially useful clinical criterion to distinguish LBD from AD and encouraging further assessment for accurate diagnosis [25, 26]. Clinical data indicated that the median survival period following diagnosis for LBD was approximately 4 years for patients, compared to 6 years for patients with AD [20, 27, 28], suggesting a potentially shorter survival duration for LBD following diagnosis compared to AD.

Pathological verification remains essential for definitive AD and LBD diagnosis. Despite established clinical criteria, differentiating AD from LBD based solely on symptoms remains challenging without pathological or biomarker support. Current diagnostic standards for DLB demonstrate initial clinical sensitivity and specificity values both ranging from approximately 70–90%, though misdiagnosis remains a significant challenge [29, 30]; therefore, biomarker profiling has emerged as a critical method for differential diagnosis. Cerebrospinal fluid (CSF) analysis reveals that concentrations of total tau protein (t-Tau) and phosphorylated tau-181 (p-Tau 181) are significantly elevated in AD patients compared to DLB counterparts [31–33], but their discriminative power may be confounded by comorbid amyloid pathology in mixed cases [34]; therefore, these measures may function as potential discriminators between AD and DLB. Simultaneously, the Aβ_42_ to Aβ_40_ ratio exhibits possible diagnostic performance in differentiating AD from DLB, with potential discriminative advantages observed during early disease progression [35, 36]. Notably, while characteristic biomarkers are detectable in most AD patients, only 15%-60% of prodromal DLB cases present typical pathological markers. Furthermore, 28%-60% of DLB patients at dementia stages still lack specific biological signatures [32, 35, 37]. These findings underscore the critical clinical value of integrating conventional diagnostic assessments with biomarker detection for accurate AD and DLB differentiation.

### PD vs. LBD

PD and LBD are neurodegenerative disorders characterized by Lewy body (LB) formation and accumulation, manifesting as progressive movement disorders [38]. In the early stages of PD, non-motor symptoms predominate, including REM sleep behavior disorder, and olfactory dysfunction [39, 40]. Motor symptoms emerge in the mid to late stages, presenting as resting tremor, rigidity, bradykinesia, gait abnormalities, and postural instability [41]. LBD patients exhibit cognitive and arousal fluctuations, visuospatial hallucinations, spontaneous parkinsonian features, REM sleep behavior disorder, and behavioral dysregulation [7]. While cognitive function may remain relatively preserved in PD until the late stages, LBD involves an earlier, more progressive decline due to diffuse Lewy body pathology affecting both cortical and subcortical regions [42].

α-syn is a pivotal protein in the pathogenesis of synucleinopathies, with both PD and LBD characterized by abnormal accumulations of α-syn leading to neuronal damage [43]. In PD, α-syn deposition is primarily restricted to the brainstem and limbic regions. In contrast, in LBD, α-syn deposition extends to the neocortex, involving additional brain regions [44]. Dopaminergic neuron loss predominantly occurs in the substantia nigra compacta (SNc) in PD, whereas LBD exhibits more extensive α-syn pathology, including brainstem, limbic, and neocortical spread of Lewy body lesions. This widespread cortical and subcortical involvement distinguishes LBD’s multisystem neurodegeneration, affecting various brain regions, from PD’s isolated SNc damage [9, 45]. These differences in pathological distribution not only highlight the distinct neurodegenerative profiles of PD and LBD but also provide critical insights for the development of diagnostic biomarkers.

While cognitive symptoms remain the primary basis for clinical differentiation between LBD and PD [9], other proteins such as Aβ, Tau, and TAR DNA-binding protein-43 (TDP-43) contribute to the unique pathological profile of LBD and may enhance diagnostic accuracy. Like Tau pathology, which is more pronounced in LBD than in PD, LBD is characterized by the presence of neurofibrillary tangles (NFTs), which are hallmark features of Tau pathology. In contrast, PD typically shows less severe Tau pathology, and when present, it is typically limited to the brainstem (e.g., nigral neurons) rather than the neocortex [46]. This distinction is critical, as Tau pathology in LBD is associated with cognitive decline and dementia [47]. Moreover, Aβ deposition and TDP-43 abnormalities (including cytoplasmic inclusions and phosphorylation) are significantly more common in LBD than PD [48, 49]; these pathologies correlate with accelerated cognitive decline and disease progression [50].

Furthermore, studies have shown that LBD patients exhibit a distinct CSF profile compared to PD patients. LBD patients often have lower CSF Aβ_42_ levels, along with higher levels of Tau and phosphorylated Tau (p-Tau), reflecting the co-occurrence of Aβ and Tau pathology [51]. In contrast, PD patients typically have normal or only mildly reduced CSF Aβ_42_ levels and less pronounced Tau elevations [52]. Positron Emission Tomography (PET) imaging studies have demonstrated that LBD patients exhibit higher Aβ deposition in cortical regions compared to PD patients, consistent with the pathological evidence [49, 53]. Moreover, Tau PET imaging in LBD reveals distinct patterns of Tau distribution compared to PD, further supporting the role of Tau in distinguishing between these disorders [54].

Comprehensively elucidating the pathogenesis of LBD is pivotal for enhancing diagnostic precision and developing effective therapeutic strategies. Presently, there remains a lack of robust treatment options for LBD, underscoring the urgency to delve deeper into the mechanistic underpinnings of this disease. Patients with LBD often present with a series of clinical features that share overlap with AD and PD, necessitating a nuanced understanding of these shared and distinct manifestations. Over half of LBD have AD co-pathology, its prevalent but not universal, with Aβ, α-syn, and Tau lesions commonly co-existing and synergistically contribute to cognitive decline, motor dysfunction, and accelerate disease progression in a subset of LBD patients [55, 56]. Therefore, a detailed elucidation of the clinical characteristics of LBD is essential for establishing accurate diagnostic criteria and improving patient management.

## Clinical features

The DLB Consortium has rigorously established the core clinical criteria and supportive features that characterize DLB. Core symptoms in affected individuals typically encompass cognitive fluctuations, recurrent visual hallucinations, spontaneous parkinsonian motor features, and REM sleep behavior disorder [7]. These core symptoms are often masked by the more apparent manifestations of AD, leading to misdiagnosis in early stages; Notably, the presence of REM sleep behavior disorder can significantly enhance diagnostic sensitivity and accuracy for DLB [57].

In addition to the core features, supportive clinical indicators aid in the diagnostic evaluation in DLB. These include hyposmia, which occurs earlier in DLB than in AD, excessive daytime sleepiness, and an increased sensitivity to antipsychotic medications due to the decreased availability of D2 dopamine receptors in patients with DLB [7, 58]. Additional supportive features may involve postural instability, frequent falls, syncope, or transient episodes of unresponsiveness [9]. Patients may also experience autonomic dysfunction, such as constipation and urinary incontinence. While orthostatic hypotension can occur during LBD, its prevalence and timing are variable and not pathognomonic for the disorder [7]. Furthermore, some patients may exhibit psychiatric symptoms such as apathy, anxiety, and depression, which can be profound and contribute to the clinical profile of DLB [7, 9].

The absence of direct biomarkers for LBD poses challenges to its diagnosis. However, certain indicative biomarkers can aid in the diagnosis of LBD. Recent studies have demonstrated that dopamine transporter (DAT) imaging using Single-Photon Emission Computed Tomography (SPECT) (e.g., ^123^I-FP-CIT) shows high diagnostic sensitivity (78%-93%) and specificity (84%-90%) for DLB, particularly in distinguishing it from AD, though its accuracy can be influenced by factors such as medication use, patient movement, and image processing, thus it should be used in conjunction with other diagnostic tools and clinical assessments to enhance diagnostic confidence [59–61]. Besides, multimodal imaging with ^123^I-FP-CIT SPECT and a higher cingulate island ratio on ^18^F-fluorodeoxyglucose PET correlates with lower Braak tangle stage, aiding in differentiating DLB from other dementias [62, 63]. Additionally, ^123^Iodine-metaiodobenzylguanidine (MIBG) myocardial scintigraphy, which reflects cardiac sympathetic denervation, has shown high sensitivity and specificity in delayed-phase imaging for DLB, with pathological studies linking the reduced MIBG uptake to α-syn deposition in the peripheral autonomic nerves, which is a hallmark of DLB pathology [64, 65].

Besides, brain blood flow perfusion and metabolic imaging using PET/SPECT scan can also provide valuable diagnostic information. DLB patients often exhibit significantly reduced blood flow and metabolism in the occipital cortex, while showing relatively preserved metabolism in the posterior cingulate cortex (PCC) [66, 67]; therefore, the occipital hypometabolism on PET imaging can effectively aid in distinguishing DLB from AD, with reported sensitivity of 71%-90% and specificity of 64%-80%, respectively [68–70].

Further, electroencephalography (EEG) has demonstrated distinct abnormalities in DLB compared to AD, characterized by reduced peak frequencies, increased slow-wave activity, and disrupted network connectivity [71]. A multicenter study analyzing EEG data from 79 DLB and 133 AD patients revealed significant differences in quantitative EEG parameters: DLB exhibited a dominant frequency (DF) < 8 Hz, elevated DF variability (DFV > 0.5 Hz), high pre-alpha frequency prevalence (FP pre-alpha > 50%), and low alpha frequency prevalence (FP alpha < 25%), whereas AD showed stable alpha DF (DF > 8 Hz), low DFV (< 0.5 Hz), and elevated FP alpha (> 55%) [72]. Multivariate discriminant analysis identified threshold values (DF = 8 Hz, DFV = 2.2 Hz, FP pre-alpha = 33%, FP alpha = 41%) that achieved 90% accuracy in classifying DLB and 64% accuracy in classifying AD, underscoring the potential of quantitative EEG as a non-invasive biomarker for DLB diagnosis [72]. Moreover, polysomnography reveals REM sleep without atonia in DLB patients, which is another potential biomarker [73]. Together, these indicative biomarkers, can provide sufficient evidence for a probable diagnosis of DLB.

It is noteworthy that the initial clinical presentations of DLB and PDD exhibit distinct characteristics [7, 74]. However, as PD progresses and dementia becomes manifest, the clinical and biological differentiations between PDD and DLB become less pronounced. Consequently, the clinical features and diagnostic criteria for these conditions exhibit considerable convergence [75].

### Pathogenesis

LBD is a heterogeneous disorder characterized by a composite of synaptic degeneration, neuronal loss, and basal forebrain cholinergic degeneration [44]. The variegated symptoms suggest a multifaceted etiology underlying LBD. Co-existing AD and PD related pathologies are observed, where amyloidogenic Aβ and hyperphosphorylated Tau contribute to cognitive decline, while α-syn aggregation leads to Lewy bodies and Lewy neurites (LNs) formation, underpinning parkinsonism and visual hallucinations in LBD [18].

### α-Synuclein

α-Syn is a central protein in the pathogenesis of synucleinopathies, a small protein consisting of 140 amino acids (14 kDa) and encoded by the *SNCA* gene [76]. α-syn is structurally divided into three domains: the amino-terminal domain (residues 1–60), the central hydrophobic domain (residues 61–95), and the acidic carboxy-terminal domain (residues 96–140) [77]. Under physiological conditions, α-syn exists primarily as an unfolded, membrane-bound monomer and may function in the regulation of vesicle fusion and neurotransmitter release at the synapse [78–80]. Studies on α-syn expression reveal its abundant levels in the developing human brain, particularly during early gestation, with a re-elevated expression pattern observed in synucleinopathies [81]. Mutant α-syn can lead to the formation of filaments, which are commonly observed and associated with familial PD, including duplication, triplication or point mutations such as A53T [82], A30P [83], E46K [84], H50Q [85], or G51D [86]. These mutations further enhance α-syn aggregation through conformational changes [87]. Mutant α-syn is often unable to be degraded and further aggregated into Lewy bodies and Lewy neurites, leading to neurodegeneration.

Abnormal α-syn aggregation is a central pathological feature of synucleinopathies. These aggregates disrupt neuronal function and propagate between neurons, spreading the pathology throughout the nervous system [88]. Specifically, α-syn aggregates impair mitochondrial function, causing energy deficits, oxidative stress, and disrupt lysosomal activity [89–91], leading to the accumulation of damaged proteins and organelles [92]. Additionally, α-syn oligomers permeabilize neuronal membranes, triggering calcium influx that disrupts intracellular homeostasis and activates cytotoxic cascades (e.g., mitochondrial dysfunction and apoptosis) [93–95]. Moreover, α-syn aggregates activate microglia, initiating neuroinflammation that releases pro-inflammatory cytokines and reactive oxygen species (ROS) to exacerbate neuronal damage [96]. Collectively, these α-syn driven mechanisms underlie progressive neurodegeneration in synucleinopathies, highlighting its pivotal role in pathogenesis.

In LBD, α-syn mutations have also been identified, with α-syn combining with ubiquitin, neurofilaments, and α-crystallin B to form inclusions [97–99]. These inclusions can deposit intracellularly and extracellularly, propagating to recipient neurons through a prion-like mechanism in PD and LBD [100]. Post-translational modifications of α-syn, including phosphorylation, ubiquitination, and nitration, have been identified [101]. In DLB, approximately 90% of insoluble α-syn is phosphorylated at S129, while only 4% of soluble α-syn is present in the cytosol [102], suggesting that phosphorylated α-syn primarily contributes to its aggregation. Clinical observations have associated Lewy body pathology with the onset of hallucinations in LBD [103], as opposed to Aβ or Tau pathology. Lewy body pathology is a key factor contributing to impairments in attention, executive function, visuospatial abilities, and motor skills, as well as to a more rapid decline in cognitive function over time [103]. Consequently, Lewy body pathology is crucial for predicting and managing cognitive impairment in LBD, offering a significant approach for clinical diagnosis and management strategies.

### Amyloid β

The production of Aβ originates from the cleavage of amyloid beta precursor protein (APP), an integral membrane protein expressed across various tissues and involved in cell surface receptor functions to regulate synaptic formation and neural plasticity [104, 105]. APP undergoes metabolism through α, β, and γ secretases [106]. α-Secretase cleavage of APP precedes γ-secretase cleavage, generating nonamyloidogenic fragments [107]. In contrast, in AD pathology, β-secretase cleavage is followed by γ-secretase, resulting in the production of various potentially amyloidogenic Aβ peptides, which range in length from 38 to 43 amino acids [108]. Aβ_1−42_ is commonly detected in AD brains [109]. While direct evidence for the presence of specific Aβ peptides in LBD patient brains are lacking, several studies have suggested that Aβ_1−38_, Aβ_1−40_, and Aβ_1−42_ are frequently exhibited in cerebrospinal fluid (CSF) [110, 111], indicating that these proteins may be present in the brain in LBD patients. Approximately half of LBD patients exhibited Aβ deposition in the neocortex, as observed in autopsy or neuroimaging studies, with Aβ_1−42_ being the primary form prone to deposition in amyloid plaques [112, 113]. Notably, Aβ plaques trigger oxidative stress and mitochondrial dysfunction, disrupting synaptic transmission and impairing cellular homeostasis [114]. They also interfere with long-term potentiation—a critical process for memory formation, thereby hindering synaptic plasticity and contributing to cognitive decline [115]. Moreover, chronic activation of microglia and astrocytes in response to Aβ accumulation releases pro-inflammatory cytokines and ROS, exacerbating neuronal damage and accelerating disease progression [116, 117]. Importantly, researches showed that Aβ interacts other proteins like α-syn, which further enhancing the aggregation of α-syn in LBD pathology. This co-pathology highlights the interplay between Aβ and synucleinopathies, underscoring the complexity of LBD as a multisystem neurodegenerative disorder. This interaction will be further discussed in the subsequent section (Aβ enhances α-syn pathology), shedding light on the interconnected mechanisms driving LBD progression.

### Tau

Tau is another key protein involved in the pathogenesis of LBD, belonging to a family of six soluble protein isoforms generated through alternative splicing of the microtubule-associated protein Tau (*MAPT*) gene [118]. Tau is prominently expressed throughout the human brain and is primarily responsible for the stabilization of microtubules within axons [118]. Exons 2, 3, 4a, 6, 8, and 10, which are the alternatively spliced cassette exons of the *MAPT* gene, can produce six major isoforms of the Tau protein [119]. The exclusion of exon 10 results in the production of 3R-Tau, which contains three microtubule-binding domain (MTBD) repeats, whereas the inclusion of exon 10 leads to the production of 4R-Tau, which harbors four MTBD repeats [120]. In LBD, Tau undergoes abnormal hyperphosphorylation, detaching from microtubules and aggregating into neurofibrillary tangles (NFTs), and these NFTs not only disrupt the structural integrity of neurons but also impede axonal transport, leading to neuronal dysfunction and cell death [121, 122]. The spread of Tau pathology across interconnected brain regions correlates with the progression of cognitive decline, making it a critical factor in the development of the disease [123]. Mutant Tau, characterized by hyperphosphorylation, forms insoluble aggregates known as neurofibrillary tangles, which are a hallmark of both AD and LBD [124, 125]; these aggregates significantly contribute to the etiology of LBD.

### Transactive response DNA-binding protein-43

Another protein that is prevalent in LBD is TDP-43, an RNA-binding protein encoded by the *TARDBP* gene [126]. TDP-43 is primarily responsible for regulating a multitude of RNA processes, encompassing splicing, translation, transport, and stability [127, 128]. Abnormal hyperphosphorylation and aggregation of TDP-43 have been documented in both neurons and glial cells [129]. Studies have demonstrated the presence of TDP-43 inclusions within neurons and oligodendroglia in the brains of patients suffering from DLB [50]. The formation and accumulation of these aggregates are thought to disrupt critical cellular functions, including RNA metabolism and protein homeostasis. Remarkably, brain regions such as the amygdala and hippocampus, maybe more vulnerable to Tau or α-syn pathology and exhibit more severe TDP-43 pathology in cases of AD and DLB [130]. Research observed the colocalization of TDP-43, aggregated α-syn and hyperphosphorylated Tau in cytoplasmic inclusions within amygdala and hippocampal neurons in AD cases [131]. Postmortem studies demonstrate significant colocalization of TDP-43 and α-syn pathology in limbic regions of DLB patients. The severity of α-syn pathology in temporal cortical areas positively correlates with TDP-43 burden in the amygdala [132]. Mechanistically, TDP-43 potentiates α-syn neurotoxicity by facilitating the formation of α-syn/TDP-43 hybrid fibrils. This co-occurrence of pathologies suggests a potential synergistic effect, where the interplay between TDP-43 aggregates and other pathological proteins exacerbates the neurodegenerative process [132]. The presence of TDP-43 pathology in these regions may contribute to the complex clinical presentations observed in AD and DLB, including cognitive decline and neuropsychiatric symptoms [133–135]. Understanding the intricate relationship between TDP-43 and other pathologies is crucial for developing effective therapeutic strategies to address the multifaceted nature of these neurodegenerative diseases.

### Neuroinflammation

Neuroinflammation has emerged as a pivotal etiological factor in LBD. PET imaging revealed microglial activation in vivo [136], while genetic investigations have identified polymorphisms in inflammation-related genes as risk factors for DLB [137]. Moreover, evidence of inflammatory responses was present during the prodromal stages of DLB, characterized by early increased in cytokines such as IL-1β, IL-2, IL-4, and IL-10 [138]. These findings underscore the important role of neuroinflammation in the pathogenesis of LBD and imply that strategies targeting neuroinflammation could be beneficial in the management of the disease.

### Mitochondrial dysfunction

Additionally, clinical assessments have revealed decreased levels of mitochondrial DNA (mtDNA) and the biogenesis markers PGC-1α and PGC-1β in post-mortem prefrontal cortex tissue from DLB and PDD patients [139], indicating mitochondrial dysfunction as a contributing factor. Moreover, mutations in PINK1/parkin, DJ-1, and LRRK2, which are known to affect mitochondrial function, have been implicated in the pathogenesis of LBD [140]. Furthermore, post-mortem brain tissue analysis has shown a significant upregulation of the UPR mediator GRP78/BiP in the cingulate gyrus and parietal cortex of DLB and PDD patients [141], underscoring the relevance of UPR activation in the etiology of LBD and its potential implications for disease pathology.

### Epigenetic change

In recent years, epigenetic change has been recognized as one of the hallmarks of LBD. Given the central role of α-syn aggregation in the pathology of Lewy body diseases, extensive investigation has been conducted into the methylation status of the *SNCA* gene [142]. Studies have observed global DNA hypomethylation, with particular emphasis on the hypomethylation of intron 1 within the *SNCA* gene in post-mortem brain tissue from DLB patients [143]. Further analysis has identified 1075 differentially methylated promoters (DMPs) in LBD, which are associated with altered gene expression patterns [144]. Moreover, research has identified 17 significantly differentially methylated CpGs (DMCs) and 17 differentially methylated regions (DMRs) from autopsy brain tissue of DLB patients, offering valuable insights into the pathogenesis of DLB and holding the potential for future utilization as biomarkers for the diagnosis of DLB [145]. Additionally, a comprehensive epigenome-wide association study (EWAS) conducted across different stages of Braak Lewy body progression in post-mortem human frontal cortex has identified four novel differentially methylated loci (TMCC2, SFMBT2, AKAP6, PHYHIP) that demonstrate replication across various sample series in DLB [146]. The interplay between genetic variability and epigenetic modifications in key genes implicated in LBD, such as apolipoprotein E (*APOE*), α-syn (*SNCA*), and glucocerebrosidase (*GBA*), underscores the pivotal role of epigenetic mechanisms in the pathogenesis of disease [147–149]. Consequently, we further focused on elucidating the genetic risk factors that contribute to the development of LBD, with the aim of enhancing diagnostic and therapeutic strategies for this condition.

### Lipid metabolism aberrant

Lipids emerge as a pivotal regulator in the α-syn aggregation [150]. Alterations in the lipid/α-syn ratio, modifications in lipid composition, and variations in the proportion of membrane bound or unbound α-syn perturb the equilibrium and facilitate the onset of α-syn fibril formation [150]. Prior research has detected a reduction in lipid metabolism in both the *C. elegans* LBD model and in the lateral temporal lobe of post-mortem brains from DLB patients [151]. Additionally, lipid rafts from DLB patients exhibit disturbed lipid profiles, characterized by reduced levels of long-chain polyunsaturated fatty acids, plasmalogens, and cholesterol, along with decreased unsaturation and peroxidability indices [152]. Disturbances in lipid metabolism are sufficient to promote the pathological aggregation of misfolded proteins (Aβ, Tau, α-syn, and TDP-43) associated with neurodegenerative diseases [153, 154]. These findings underscore a profound link between lipid dysregulation and the pathogenesis of LBD, emphasizing the importance of lipid homeostasis in neurodegenerative processes.

### Others

**Fig. 1:**
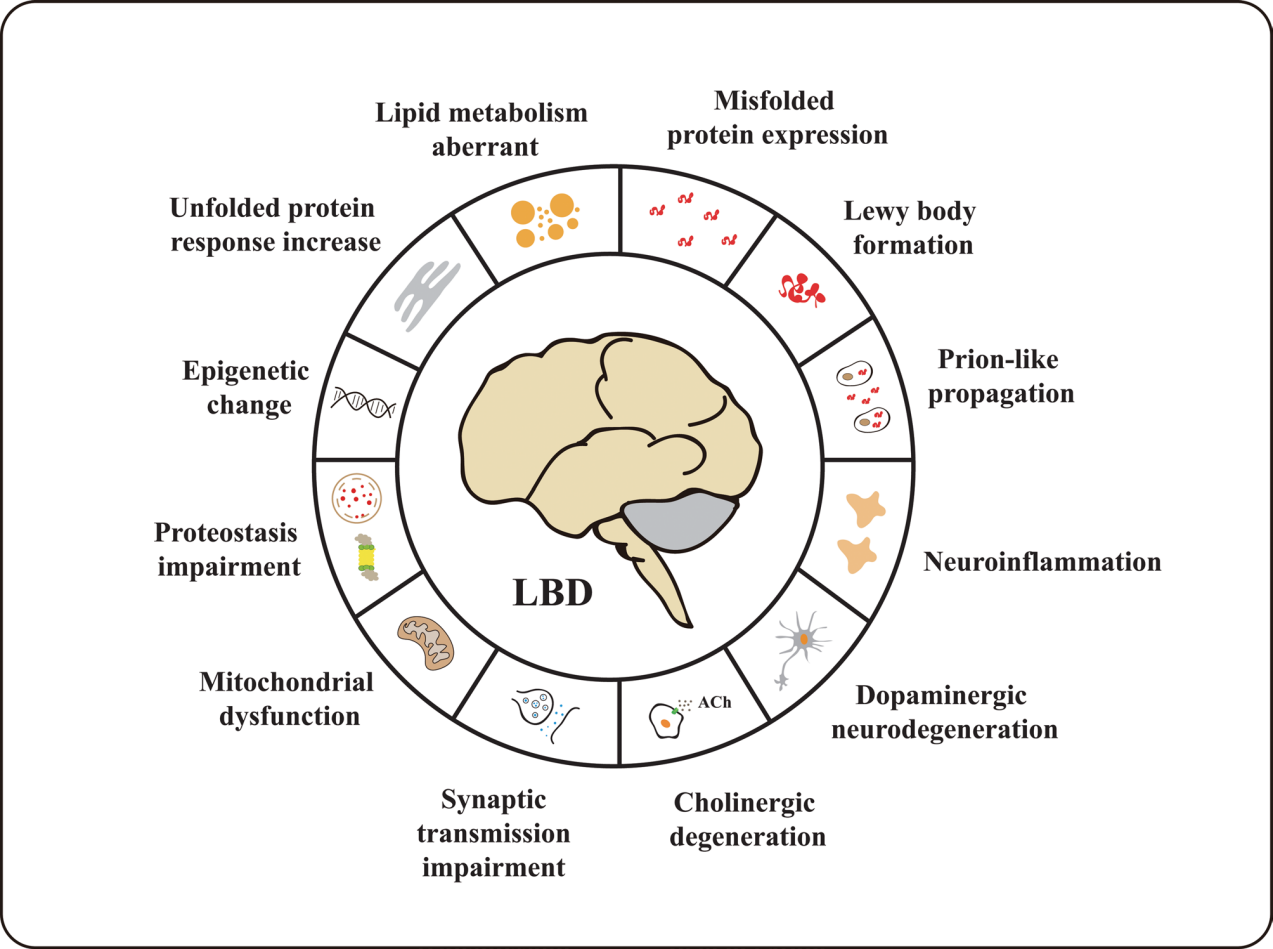
The pathogenesis hallmarks of LBD

Other mechanisms also drive LBD pathophysiology. Clinical PET imaging studies have shown that synaptic densities are widely decreased in the cortex of LBD patients [155], highlighting synaptic loss as a crucial mediator of cognitive dysfunction. Moreover, dopaminergic neurodegeneration [156], cholinergic degeneration [157], proteostasis impairment [158], and an increase in the unfolded protein response (UPR) [141] also play roles in the pathogenesis of LBD (Fig. 1). These interconnected pathologies contribute to the unique clinical manifestations of LBD and present therapeutic challenges that demand innovative approaches for effective management.

In summary, the complex interplay among various misfolded proteins, including Aβ, α-syn, Tau, and TDP-43, alongside with the involvement of multiple pathological processes such as neuroinflammation, synaptic and mitochondrial impairment, dopaminergic and cholinergic degeneration, lipid metabolism aberrations, proteostasis impairment, unfolded protein response increase and epigenetic changes (Fig. 1), underlies the pathogenesis of LBD, which in turn gives rise to its diverse clinical manifestations and therapeutic challenges. This understanding paves the way for further research into potential therapeutic interventions and diagnostic biomarkers to better manage this debilitating disorder.

### Genetic risk factors

Aging is a predominant risk factor for the onset of LBD. With the rise of global aging population, there is a correspondingly substantial increase in the prevalence of LBD [159]. Genetic risk factors further contribute to the incidence of LBD, with a positive family history increasing the risk of disease onset by 2.3-fold among siblings [160]. Genome-wide association studies (GWAS) have investigated samples from 22 centers across 10 countries, encompassing 1743 LBD patients and 4454 controls, to pinpoint factors potentially influencing LBD [161]. These findings confirm *APOE*, *SNCA*, and *GBA* as genetic risk factors, with approximately 36% of the LBD risk being attributed to heritable factors [161]. Subsequent studies on Norwegian and European cohorts, which included a total of 828 DLB cases and 82,035 controls, further confirmed that variants in the* GBA* and *APOE* ε4 loci are strongly associated with the incidence of LBD [149].

### APOE

The *APOE* gene, which resides on chromosome 19q.13.2, is a pivotal gene encoding a major lipid transporter in brain [162]. *APOE* gene encodes three principal isoforms: APOE2 (derived from the ε2 alleles), APOE3 (derived from the ε3 alleles), and APOE4 (derived from the ε4 alleles). These isoforms arise from variations at two non-synonymous nucleotide polymorphisms within *APOE* exon 4: rs429358 (C/T) and rs7412 (C/T), which result in cysteine-to-arginine mutations at positions 112 and 158 in the protein sequence, respectively [163]. These amino acid substitutions alter the structural stability, lipid-binding affinity, and receptor interactions of *APOE*, leading to isoform-specific effects on lipid metabolism, neuroinflammation, and protein aggregation [164–167]. A substantial body of research has identified *APOE*, particularly *APOE4*, as a significant genetic risk factor for LBD [149, 161]. Prior studies have shown that *APOE4* allele is associated with an increased risk of LBD in cases with moderate or high AD pathology, and even in LBD subgroups exhibiting low AD pathology, *APOE4* carriers show a higher prevalence of Lewy bodies [168]. Notably, *APOE4* carriers also experience increased mortality rates in LBD, further highlighting its detrimental role in disease progression [20]. These may be attributed to propensity of *APOE4* which promote α-syn aggregation, disrupt endosomal-lysosomal trafficking, and impair lipid metabolism, and all of these factors contribute to neurotoxic protein accumulation [165, 169, 170].

**Fig. 2:**
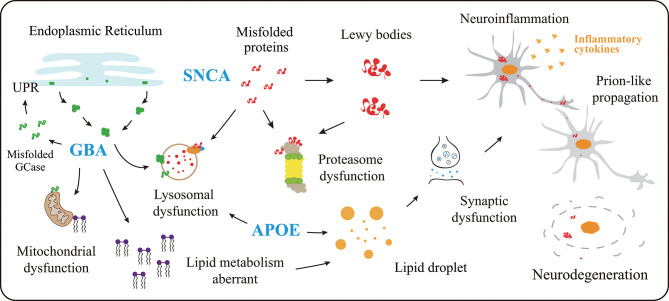
Genetic risk factors contribute to the pathogenesis of LBD. *APOE* induces structural alterations in lipid-binding domains, reduces lipidation efficiency, and promotes self-oligomerization, thereby impairing lipid transport capacity and exacerbating cellular stress. These conformational changes may disrupt lipid homeostasis, contributing to synaptic dysfunction and elevated susceptibility to Lewy body formation. *SNCA* encodes α-syn protein, while mutant α-syn aggregation impairs proteostasis systems through autophagy inhibition and proteasome dysfunction, thereby exacerbating toxic protein accumulation and inducing cellular stress. Pathological α-syn further activates microglia-mediated neuroinflammation, accelerating neurodegeneration processes. *GBA* mutations lead to misfolded glucocerebrosidase (GCase) proteins that accumulate in the endoplasmic reticulum (ER), triggering ER stress and activating the unfolded protein response (UPR). Reduced GCase activity disrupts sphingolipid homeostasis, resulting in glucosylceramide and glucosylsphingosine accumulation, lysosomal dysfunction, and mitochondrial impairment. This metabolic disruption increases oxidative stress, energy deficits, and α-syn aggregation, creating a pathological feedback loop in LBD

Moreover, APOE4 pathogenic role extends beyond its association with amyloid pathology, as it exacerbates α-syn pathology through mechanisms independent of Aβ accumulation [171, 172]. Mechanistically, APOE4 induces structural changes in lipid-binding domains, reduces lipidation efficiency, and enhances self-oligomerization, leading to dysfunctional lipid transport and increased cellular stress (Fig. 2) [173]. These effects are particularly detrimental in neurons and astrocytes, where APOE4 impairs fatty acid sequestration into lipid droplets and disrupts neuron-astrocyte metabolic coupling, further amplifying synaptic dysfunction and neurodegeneration [174, 175]. This heightened risk is compounded by APOE4-driven neuroinflammation, characterized by microglial activation, and impaired clearance of pathological proteins [176–178]. Besides, APOE is critical for lipid transport in the brain. APOE4 may impair lipid homeostasis, leading to synaptic dysfunction and increased vulnerability to Lewy body formation [173].

### SNCA

*SNCA* is a gene located on human chromosome 4q21 and encodes the protein α-syn [179]. Single nucleotide polymorphisms within the *SNCA* gene are strongly associated with the formation of Lewy bodies and contribute to an increased risk of familial PD [180]. Missense mutations in *SNCA*, such as A53T, A30P, E46K, H50Q, and G51D, have been identified as risk factors that promote the aggregation of α-syn and the formation of Lewy bodies, these aggregates disrupt neuronal function and leading to the neurodegeneration in PD and LBD [181]. The abnormal aggregation of α-syn, driven by these genetic mutations, leads to multiple downstream pathological consequences. Initially, α-syn aggregates impair proteostasis systems through autophagy inhibition and proteasome dysfunction, exacerbating intracellular accumulation of toxic protein species and inducing cellular stress [182–184]. Furthermore, mitochondrial dysfunction and oxidative/nitrosative stress ensue, triggering both cell-autonomous and non-cell-autonomous neuronal death [185, 186]. Finally, pathological α-syn activates microglia-mediated neuroinflammation via innate and adaptive immune pathways, accelerating neurodegeneration (Fig. 2) [187–189]. These processes interact with each other and collectively drive the progression of PD and LBD. Studies have investigated that a polymorphic CT-rich splice variant within intron 4 of the *SNCA* gene is associated with the risk of Lewy body pathology in AD and affects the expression of *SNCA* [190]. Consequently, the *SNCA* gene serves as a significant genetic risk factor closely related to the pathology of LBD.

### GBA

The *GBA* gene is located on chromosome 1q21 and encodes glucocerebrosidase (GCase), a lysosomal enzyme responsible for glucosylceramide metabolism [191]. Mutations in the *GBA* gene have been implicated in the increased susceptibility to PD and, notably, in the heightened risk of LBD [192]. *GBA* is implicated in lysosomal dysfunction, which may exacerbate α-syn accumulation and neurodegeneration in LBD [193]. Individuals with LBD harboring *GBA* mutations face a risk that is 6 to 8 times greater than that of non-carriers, and approximately half of *GBA* mutation carriers develop dementia [194, 195]. Studies have revealed that among DLB patients, approximately 8.28% of patients carry *GBA* mutations, and these mutation carriers exhibited significantly earlier disease onset and death compared to noncarriers. Specifically, mutation carriers had a mean age at onset of 63.5 years versus 68.9 years in noncarriers [195]. The N370S and L444P variants account for 50% of *GBA* mutations [192]. Additional variants within the *GBA* gene include M123T, E326K, R496H, G202R, I260T, T369M, W393R, D409H, 84insGG, RecNciI, and novel mutations such as L144V, S488T, and P175P [192, 195, 196]. Mechanistically, *GBA* mutations disrupt sphingolipid homeostasis via reduced GCase activity, leading to glucosylceramide and glucosylsphingosine accumulation and lysosomal impairment. These metabolites promote α-syn misfolding and aggregation, fostering toxic oligomers and Lewy pathology [197]. Concurrently, lysosomal dysfunction impairs autophagy-lysosome pathway activity, compromising α-syn clearance and exacerbating its pathological accumulation [198–200]. Toxic α-syn species further disrupt lysosomal biogenesis, establishing a self-amplifying feedback loop that drives neurodegeneration (Fig. 2) [201–203]. The bidirectional interplay between α-syn aggregation and lysosomal impairment highlights *GBA* mutations as a pivotal modifiable risk factor in LBD.

Pathogenic mechanisms stemming from *GBA* mutations are multifaceted and interdependent. Misfolded GCase proteins accumulate in the endoplasmic reticulum (ER), triggering ER stress and activation of the unfolded protein response (UPR), which disrupts proteostasis and may initiate apoptosis [204, 205]. Reduced GCase activity also disrupting mitochondrial quality control, thereby promoting oxidative stress, energy deficits, and α-syn aggregation [197, 206]. Accumulated glucosylceramide and glucosylsphingosine alter membrane lipid composition, may further enhancing α-syn pathology [204, 207–209]. These disruptions activate neuroinflammatory responses via microglial engagement, perpetuating neuronal damage through pro-inflammatory cytokine release (Fig. 2) [205]. These mechanisms create a synergistic cascade wherein GCase deficiency, α-syn toxicity, mitochondrial dysfunction, and neuroinflammation converge to drive neurodegeneration. Collectively, these findings indicate that *GBA* mutations are an important modifiable risk factor for the development of LBD, particularly in a subset of patients.

### Others

Beyond the commonly known AD and PD pathogenesis associated genes of *APOE*, *SNCA*, and *GBA*, LBD has a distinct genetic architecture [161]. Studies have identified additional genes like *CNTN1*,* SCARB2*,* MAPT*,* LRRK2*,* SNCB*,* PSEN1*,* PSEN2*,* GRN*,* PARK2*,* PINK1*,* APP*,* GABRB3*,* BCL7C/STX1B*,* TREM2*,* CHMP2B*,* SQSTM1*,* EIF4G1*,* GIGYF2*,* BIN1*, and *TMEM175* as potential genetic risk factors for LBD [161, 210–212]. Although the precise mechanisms by which these genetic risk factors contribute to the pathogenesis of LBD remain elusive, it is plausible that they may adversely affect signal transduction pathways, thereby exacerbating protein misfolding and aggregation, which can ultimately result in neurodegeneration. Future experimental investigations are essential to elucidate the intricate interplay between genetic risk factors and the pathobiology of LBD.

Genetic risk factors in *APOE*, *SNCA*, and *GBA* increase the risk of LBD, while the intricate interplay among Aβ, α-syn, Tau, and TDP-43 in LBD pathogenesis underscores the need for experimental systems to validate these molecular interactions and their downstream effects. Animal models have emerged as indispensable tools to recapitulate key pathological features of LBD, enabling researchers to dissect the causal relationships between protein aggregation, neuroinflammation, and neurodegeneration. These models not only bridge the gap between neuropathological observations and clinical manifestations but also provide a platform for testing therapeutic interventions. The following section synthesizes findings from animal studies, highlighting their pivotal role in elucidating the molecular underpinnings of LBD and guiding future translational research.

### Animal models for LBD

Most reviews have collated clinical case studies on LBD, acknowledging the elusive etiology of the disorder and the limitations of conducting experiments on LBD patients. These constraints have highlighted the indispensable role of animal models in deciphering LBD’s molecular architecture. Clinical evidence reveals the co-occurrence of Aβ, α-syn, Tau, and TDP-43 proteins in LBD patients [213, 214]. Specifically, neuropathologically confirmed cases demonstrate approximately half of co-occurrence of Aβ and α-syn pathologies, a quarter displaying Tau and α-syn co-existence, and a minority showing TDP-43 and α-syn co-occurrence [213]. To investigate the interplay and mechanisms among these proteins, researchers have engineered animal models that expressed Aβ, α-syn, Tau, and TDP-43, with the aim of revealing the dynamic interactions that drive pathological transformation in LBD (Table 1). This review synthesizes findings from LBD animal models, emphasizing their pivotal role in elucidating the molecular foundations of the disease.

### Aβ enhances α-syn pathology

Given the observed co-occurrence of Aβ and α-syn in LBD, researchers have focused on exploring potential synergistic interactions between these proteins. Pioneering studies have engineered transgenic mouse models that neuronally expressed human Aβ and α-syn, further highlighting the exacerbation of α-syn accumulation by Aβ co-expression [215]. Subsequent in vivo studies have examined the brain samples from LBD patients and observed extensive accumulation of α-syn oligomers, including dimers, trimers, pentamers, and higher-order aggregates [216]. These findings have been corroborated by studies using transgenic mice that co-expressed the amyloid precursor protein (APP) and α-syn, which demonstrated that Aβ increased the levels of α-syn oligomers [216]. Additionally, research has indicated that reducing endogenous α-syn expression in an APP transgenic mouse model can prevent the degeneration of cholinergic neurons and ameliorate behavioral deficits, suggesting that decreased α-syn expression mitigates Aβ toxic effects [217].

Molecular modeling and simulation studies have provided further insights into the interaction between Aβ and α-syn; results suggested that Aβ bound to α-syn monomers, homodimers, and trimers, facilitating the formation of hybrid ring-like pentamers [216]. Predominantly, these interactions occurred at the N-terminus of Aβ and at both the N-terminus and C-terminus of α-syn. The complexes formed between Aβ and α-syn appear to be more stable, resulting in the formation of pentamers and hexamers adopting a ring-like structure that exhibits increased resistance to degradation [216]. This research enhanced our understanding of the interplay between Aβ and α-syn underlying the pathogenesis of LBD.

While the precise roles of Aβ and α-syn in inducing interaction and exacerbating aggregation remain ambiguous, significant research has focused on determining their seeding effects. Investigations have explored whether fibrils or cross-linked oligomers of Aβ_1−40_, Aβ_1−42_, and α-syn exhibited cross-seeding capabilities on aggregation [218]. In vitro findings have suggested that the hydrophobic core of α-syn fibrils possessed a stronger seeding effect than Aβ_1−40_ and Aβ_1−42_ [218]. In contrast, research has uncovered that monomeric and fibrillar α-syn exhibited opposite effects on Aβ aggregation [219]. Monomeric α-syn has been observed to inhibit the autocatalytic propagation of Aβ_1−42_ fibrils, whereas fibrillar α-syn promoted the heterogeneous nucleation of Aβ_1−42_ aggregation [219]. These observations indicated that the fibrillar form of α-syn may serve as a facilitator for enhancing Aβ aggregation.

To further validate the synergistic interplay between Aβ and α-syn, researchers injected exogenous α-syn fibrils into mice already expressing abundant Aβ plaques [220]. The results revealed that Aβ deposits significantly expedited the pathogenesis of α-syn and led to its widespread dissemination throughout the mouse brain. These findings suggested a feedback mechanism, where Aβ plaques enhanced α-syn seeding and spreading [220]. Our research group also explored the protein interactions by neuronally expressing human Aβ and α-syn(A53T) in a *C. elegans* model [151]. We observed that the concurrent expression of these two proteins exacerbated deficits in thrashing, egg laying, serotonin, and cholinergic signaling, as well as dopaminergic neuron function. Additionally, Aβ was found to increase α-syn expression in the transgenic worms [151]. Collectively, these research findings indicated that Aβ promoted the aggregation of α-syn.

One of the key features of LBD is the degeneration of synapses and the concomitant decline in synaptic protein levels [221, 222]. To delve into the molecular underpinnings of synaptic damage accompanying the co-occurrence of Aβ and α-syn in cultured cortical and hippocampal neurons, researchers have analyzed the presynaptic membrane protein synaptophysin [223]; results revealed that α-syn reduced synaptophysin levels and impaired synaptic vesicle recycling, a process that was exacerbated by the presence of Aβ, suggesting a synergistic effect on synaptic dysfunction in LBD [223]. In exploring the signaling pathways that underlie the co-existence of Aβ and α-syn pathologies, studies using neuronal cells with overexpressed α-syn have uncovered that Aβ inhibited autophagy and further enhanced α-syn aggregation [224]. Furthermore, Aβ induced mitochondrial dysfunction and activated oxidative stress signaling pathways by downregulating the AMPK/Sirt1 signaling axis in cells with increased α-syn expression [224]. Additionally, Aβ has been demonstrated to impair insulin sensitivity, leading to the downregulation of the nuclear factor erythroid 2-related factor (NRF2)/heme oxygenase 1 (HO-1) antioxidant signaling pathway, which promoted α-syn aggregation in turn [225].

Further research indicated that α-syn compromised the ubiquitin-proteasome system and upregulated the expression of apolipoprotein E (ApoE), thereby contributing to Aβ accumulation [176]. While knockout of ApoE had been shown to delay neurodegeneration in the Aβ and α-syn co-expressed transgenic mice, suggesting that extracellular signaling pathways involving ApoE play a critical role in mediating the interaction between Aβ and α-syn [176]. Moreover, investigations had revealed that the vulnerability of hippocampal neurons to the toxic effects of α-syn and Aβ may be mediated through the metabotropic glutamate receptor 5 (mGluR5) [226]. Aβ oligomers was found to increase intracellular calcium levels, activate calpain, and promote caspase-3-dependent cell death, which can be mitigated by inhibiting mGluR5 in neuronal cells overexpressing α-syn [226]. These findings imply that therapeutic interventions targeting ApoE or mGluR5 could represent viable strategies for the treatment of LBD.

### Tau binds with α-syn

The density and distribution of neurofibrillary tangles, driven by hyperphosphorylated Tau, are typically lower in LBD compared to AD [227]. Both hyperphosphorylated Tau and Lewy bodies are present in the neocortex, with instances of colocalization of these two proteins within the same Lewy body deposits having been observed [228–230]. Furthermore, clinical studies had identified the co-aggregates between Tau and α-syn in astrocytes within the middle temporal gyrus in LBD cases [230]. Consequently, a significant body of research focuses on elucidating the intricate interplay between these two proteins.

In vitro investigations had provided insights into the complex interplay between Tau and α-syn, with foundational research having identified interaction complexes between these proteins through affinity chromatography, suggesting a direct binding of these two proteins, and α-syn induced Tau fibrillization [231]. Further, studies had elucidated that the C-terminal region of α-syn and the microtubule-binding domain of Tau were the sites of interaction, whereas N-terminal and C-terminal fragments of Tau did not bind to α-syn [231]. Phosphorylation of Tau at serine 214 had been shown to increase the binding competence of α-syn, whereas phosphorylated α-syn at serine 129 did not exhibit this enhancing effect [232]. Analysis of mutant forms of α-syn via co-immunoprecipitation had revealed that the A30P, A35T, and E46K mutations potentiated Tau binding, whereas the E83P mutation inhibited such binding activity [233]. Notably, the P301L mutant of Tau appeared to reduce its interaction with α-syn in vitro [234]. Consequently, the precise mechanisms underpinning the interaction between Tau and α-syn require further experimental clarification and continued investigation.

To investigate the potential interaction between Tau and α-syn in vivo, researchers had identified hyperphosphorylated Tau in the brainstem and striatum in α-syn overexpressing mice, which contributed to cognitive function deficits in mouse models [235]. Additionally, *Drosophila* models that overexpress Tau and α-syn can exacerbate a rough eye phenotype and lead to the loss of dopaminergic neurons [236]. The interaction between Tau and α-syn disrupted cytoskeletal organization and axonal transport, culminating in abnormal synaptic organization that contributed to neuronal dysfunction and death [236]. In contrast, the genetic ablation of endogenous Tau in an α-syn(A53T) overexpressing mouse model had shown to ameliorate cognitive dysfunction and concurrent synaptic deficits [237]; therefore, these animal model results collectively reinforce the hypothesis that the interplay between Tau and α-syn is a pivotal player in the pathophysiology of LBD.

### Aβ, tau, and α-syn co-occurrence

Clinical patients exhibit a concurrent presence of Aβ, Tau, and α-syn in the brain, characteristic of the most severe subtypes of LBD [214]. To investigate the potential synergistic interaction arising from the co-existence of Aβ, Tau, and α-syn, researchers introduced the α-syn(A53T) transgene into an AD transgenic mouse model that already demonstrated amyloid plaques and neurofibrillary tangles due to Aβ and Tau accumulation [238]. Research findings suggested that these proteins may interact synergistically, enhancing aggregation, phosphorylation, and accumulation of one another, thereby accelerating cognitive decline in mice [238]. Moreover, a separate study injected α-syn fibrils into mice with substantial Aβ expression in the brain and found that Aβ deposits largely accelerated the pathogenesis of α-syn and its dissemination throughout the entire brain [220]. Additionally, α-syn was found to induce hyperphosphorylation of Tau; therefore, the co-occurrence of Aβ plaques, hyperphosphorylated Tau, and aggregative α-syn further exacerbated neuronal loss, which, in turn, impaired cognitive and motor performance [220]. Our group also utilized neuronal expression of human Aβ, α-syn(A53T) and Tau in *C. elegans* LBD model. We identified the movement, reproduction, memory, and neuronal degeneration defects, alongside miRNA dysregulation in *C. elegans*, indicating different pathological proteins interaction further exacerbate LBD pathology [239].

The precise mechanisms of cross-seeding among Aβ, Tau, and α-syn are still under investigation, with current theory proposing a two-step process [240]. Initially, Aβ plaques lead to the accumulation of α-syn within synaptic terminals, disrupting normal clearance and trafficking pathways. Subsequently, Aβ plaques are involved in the disassembly of microtubules, which are crucial for the maintenance of dystrophic axonal structures [241]. The mis-localization of α-syn and Tau proteins resulting from these effects can promote their interactions, potentially contributing to the propagation of pathological protein aggregates [242]. Additionally, Aβ plaques had been found to amplify the seeding activity of α-syn, which in turn induced the cross-seeding effects on Tau protein [220]. However, the underlying mechanisms of this cross-seeding in LBD warrant further exploration. Clarifying these complex interactions is essential for understanding the pathogenesis and developing novel therapeutic strategies for LBD.

In addition to Aβ, Tau, and α-syn, other proteins such as TDP-43 also implicated in the pathogenesis of LBD [130]. Research had demonstrated that mutant forms of TDP-43 exacerbated α-syn toxicity, leading to the impairment of dopaminergic neurons in mice [243]. Furthermore, our research group previously discovered that silencing endogenous TDP-43 (the homolog of *tdp-1* in *C. elegans*) effectively reduced the levels of α-syn expression in α-syn(A53T) overexpressing worms [244]. These findings suggest that reducing the interaction between misfolded proteins may mitigate the deleterious effects of neurodegeneration. Table 1 summarizes the key animals models relevant to LBD pathogenesis.

Together, animal models have significantly contributed to the elucidation of the pathogenesis underlying LBD, yet the exploration of molecular mechanisms and treatment strategies for LBD remains a critical area of research. The development of novel therapeutic approaches or pharmacological agents necessitates comprehensive investigation and validation in future studies.

**Table 1:** Animal models reveal LBD pathogenesis

Year	Genetic	Species	Mechanism	References
2001	Aβ, α-syn	Mice	Aβ facilitated α-syn aggregation and accumulation, potentially contributing to Lewy body diseases by enhancing α-syn-mediated neurotoxicity.	[215]
2003	α-syn, Tau	Mice	α-Syn promoted Tau fibrillization, and their co-aggregation synergistically enhanced fibrillization of both proteins, facilitating pathological inclusions formation.	[245]
2005	α-syn, Tau	Yeast	Co-expression of Tau with α-syn enhanced α-syn toxicity.	[246]
2005	α-syn, Tau	Mice	Mice overexpressing α-syn(A30P) exhibited aggregated α-syn, phosphorylated Tau, along with elevated levels of phosphorylated c-Jun N-terminal kinase (JNK).	[247]
2005	α-syn, Tau	HEK293 cells and neuronal cells	α-Syn interacted with the wild type Tau protein, whereas the P301L Tau mutation inhibited this binding activity; besides, the Tau(P30L) mutation significantly altered the localization of α-syn protein.	[234]
2006	α-syn, Tau	Mesencephalic neurons and mice	MPTP treatment induced distinct alterations in phosphorylated Tau and α-syn levels, promoting their co-immunoprecipitation in a cytoskeleton-free fractions and within neuronal soma.	[248]
2008	Aβ, α-syn	Mice	α-Syn-induced neurodegeneration involved the activation of the ubiquitin-proteasome system, a significant increase in apolipoprotein E (ApoE) levels, and the accumulation of insoluble Aβ in the mouse brain.	[176]
2008	Aβ, α-syn	Mice	Aβ directly interacted with α-syn and stabilized the formation of hybrid nanopores that altered neuronal activity.	[216]
2009	α-syn, Tau	SH-SY5Y, mesencephalic neurons and mice	GSK-3β activation required α-syn and formed a complex with Tau. GSK-3β inhibition attenuated MPTP-induced toxicity, reduced cell death, and decreased p-GSK-3β levels, p-Tau accumulation and α-syn aggregation.	[249]
2010	Aβ, α-syn	Cortical and hippocampal neurons	Aβ_1−42_ and α-syn synergistically impaired synaptic vesicle recycling and exacerbated synapse damage.	[223]
2010	Aβ, Tau, α-syn	Mice	Aβ, Tau, α-syn interacted in vivo to promote the mutual aggregation and accumulation of each other and to accelerate cognitive dysfunction.	[238]
2011	α-syn, Tau	Cells	α-syn fibrils cross-seeded with intracellular Tau, catalyzing the formation of Tau-based neurofibrillary tangle-like aggregates.	[250]
2011	α-syn, Tau	Cells	Tau colocalized and interacted with α-syn aggregates, and Tau overexpression enhanced the aggregation of insoluble α-syn. Co-transfection of Tau enhanced α-syn secretion and cellular toxicity.	[251]
2011	α-syn, Tau	Cells and mice	MPTP treatment increased intracellular α-syn levels, triggered Tau phosphorylation, and induced apoptosis. α-Syn knockdown reduced Tau phosphorylation, while co-expression of α-syn mutations enhanced Tau phosphorylation, causing microtubule instability.	[252]
2011	α-syn, Tau	Mice	Transgenic mice overexpressing α-syn exhibited increased levels of p-Tau and α-syn, along with elevated p-GSK-3β. These proteins colocalized in inclusion bodies resembling Lewy bodies. The mice demonstrated reduced brain volume, indicating significant atrophy.	[235, 253]
2011	α-syn, Tau	Mice	Elevated levels of α-syn and hyperphosphorylated Tau, coupled with microtubule destabilization, were primarily accumulated in the brainstem and striatum.	[254]
2011	α-syn, Tau	Mice	Transgenic mice with α-syn(E46K) overexpression exhibited extensive neuronal Tau inclusions, resembling neurofibrillary tangles; these mice manifested age-dependent motor deficits.	[255]
2011	α-syn, TDP-43	Mice	Co-expression of wild type TDP-43 and mutant α-syn in transgenic mice led to a more severe loss of dopaminergic neurons; TDP-43 enhanced the toxicity of α-syn toward dopaminergic neurons in vivo.	[243]
2013	α-syn, Tau	Mice	Distinct synthetic α-syn fibrils varied significantly in their efficiency in seeding Tau aggregation.	[256]
2013	α-syn, Tau	Yeast	Co-expression of α-syn and Tau led to a synergistic toxic effect by increasing intracellular α-syn inclusions formation and enhancing Tau phosphorylation and aggregation. In yeast, α-syn elevated Tau insolubility and phosphorylation through RIM11/GSK-3β.	[257]
2013	Aβ, α-syn	Neurons	High concentrations of Aβ_1−42_ and α-syn exerted toxicity toward mature neurons, while low concentrations stimulated their reciprocal production, potentially through PI3K-mediated pathway.	[258]
2013	Aβ, α-syn	Neurons	α-Syn protected primary cortical neurons from soluble Aβ_1−42_ induced cell death through the PI3K/Akt pathway. Recombinant α-syn directly interacted with Aβ_1−42_ and reduced Aβ_1−42_ oligomer levels, thereby exerting neuroprotection.	[259]
2014	Aβ, α-syn	SH-SY5Y neuroblastoma cells	Aβ exposure significantly increased the proportion of α-syn that was phosphorylated at Ser129.	[260]
2014	Aβ, α-syn	Mice	Aβ oligomers promoted an increase in intracellular calcium levels, calpain activation, and α-syn cleavage, which collectively resulted in caspase-3-dependent cell death.	[226]
2014	α-syn, Tau	*Drosophila*	α-syn and Tau interaction disrupted cytoskeletal integrity, impaired axonal transport, and damaged synaptic structure, collectively contributing to neuronal dysfunction and mortality.	[236]
2016	Aβ, α-syn	Neuronal cells	Aβ impaired autophagy, exacerbated α-syn aggregation, and dysregulated Sirt1/AMPK signaling, leading to increased ROS and mitochondrial dysfunction.	[224]
2016	Aβ, α-syn	Mice	Reducing endogenous α-syn in an APP transgenic mice prevented cholinergic neuron degeneration, improved associated functional deficits, and restored Rab3a/Rab5 protein levels.	[217]
2018	Aβ, α-syn	Neuronal cells	Aβ induced AMPK inhibition and insulin resistance, leading to the suppression of NRF2/HO-1 antioxidant pathways, which promoted α-syn aggregation.	[225]
2018	α-syn, Tau	Mice	Injection of PD-derived α-syn/Tau oligomers into Tau transgenic mouse brains accelerated endogenous Tau oligomers formation and elevated neuronal death.	[261]
2018	α-syn, Tau	Mice	Treatment of α-syn(A53T) transgenic mice with the Tau oligomer-specific monoclonal antibody reduced toxic Tau oligomer levels, protected against cognitive and motor deficits, and preserved dopamine and synaptic proteins.	[262]
2019	α-syn, Tau	Mice	Knockout of the endogenous Tau gene in α-syn(A53T) transgenic mice fully rescued cognitive impairment and synaptic dysfunction, without altering α-syn expression or the accumulation of toxic α-syn oligomers.	[237]
2019	Aβ, α-syn	Mice	Immunotherapies targeting Aβ and α-syn effectively reduced α-syn levels, improved pyramidal neuron integrity, and attenuated neuroinflammation. The synergistic benefits of combined Aβ/α-syn immunotherapy indicate a promising strategy for LBD treatment.	[263]
2020	Aβ, α-syn	Mice	Aβ accelerated α-syn pathology and dissemination in the brain, and its co-pathology with α-syn further enhanced endogenous Tau hyperphosphorylation, thereby accelerating the onset of cognitive and motor deficits and neuronal loss.	[220]
2020	α-syn, TDP-43	*C. elegans*	Knockout of *tdp-1*/TDP-43 ameliorated posture defects, movement impairments, and developmental delays in α-syn(A53T) transgenic worms and attenuated dopaminergic neuron loss.	[244]
2020	α-syn, Tau	Mice	Transgenic α-syn(A53T) mice display enhanced epileptiform activity, with myoclonus observed in half of the animals, and electroencephalogram slowing; all of these phenotypes were mitigated by Tau ablation.	[264]
2020	α-syn, Tau	Neurons	Tau aggregates cross-seeded with distinct α-syn oligomeric strains exhibited unique characteristics, like Tau aggregates seeded with dopamine-modified α-syn oligomers enhanced intracellular seeding propensity.	[265]
2020	α-syn, Tau	Mice	Overexpression of human P301L mutant Tau in transgenic mice induced phosphorylation and dimerization of endogenous α-syn by activating GSK-3β.	[266]
2020	α-syn, Tau	Mice	Human α-syn fibrils effectively cross-seeded human Tau and exhibited limited seeding efficiency toward mouse α-syn, while human Tau fibrils triggered human α-syn pathology. Combined application of these seeds showed no additive or synergistic effects.	[267]
2021	Aβ, α-syn	*C. elegans*	Co-expression of pan-neuronal Aβ and α-syn(A53T) further exacerbated thrashing deficits, egg laying impairment, serotonin and cholinergic signaling dysfunction, and dopaminergic neuron damage in *C. elegans*. Additionally, Aβ increased α-syn expression in transgenic worms.	[151]
2021	Aβ, α-syn	Mice	Aβ plaques enhanced the propagation and deposition of α-syn pathology, and this effect was correlated with the increased in neuroinflammation.	[268]
2022	α-syn, Tau	Mice	Tau facilitated α-syn aggregation and exacerbated α-syn pathology, intensifying motor and cognitive deficits. Conversely, Tau knockout reduced α-syn pathology propagation.	[269]
2022	α-syn, Tau	Mice	Loss of Tau expression markedly delayed the onset of motor deficits and decrease α-synucleinopathy progression in α-syn(A53T) transgenic mice.	[270]
2024	Aβ, α-syn, Tau	*C. elegans*	Co-expression of pathological proteins caused motor incoordination, reduced egg-laying, altered neurotransmitter signaling, cognitive impairments, and dopaminergic neurodegeneration.	[239]

### Reflection in animal model researches

Animal models have indeed revealed a spectrum of pathogenic mechanisms and signaling alterations inherent to LBD, which have significantly advanced our understanding of these complex conditions. However, it is crucial to acknowledge the limitations and challenges associated with their usage.

Firstly, animal models, including mice, *Drosophila*, *Zebrafish*, and *C. elegans*, despite harboring homologous genes for Aβ, Tau, and α-syn, do not naturally exhibit the misfolding and aggregation of these proteins into Aβ plaques, Tau neurofibrillary tangles, or Lewy bodies. Consequently, researchers have resorted to overexpressing these disease-related genes, which are mainly derived from the human genome, into animal models [151, 215, 238]. However, it is critical to acknowledge that discrepancies may exist in terms of genes transcription, translation, and post-translational modifications between different species.

Secondly, the overexpression of these misfolded genes, usually facilitated by strong promoters and multiple copies integrated into the animal genome, leads to constitutive and excessive protein production [151, 220]. This may not accurately mirror the tissue-specific expression patterns and expression levels observed in the human brain. Such discrepancies could potentially obscure the interpretation of experimental results and the translation of findings to human disease.

Moreover, variations in the utilization and research outcomes of transgenic animals across diverse cultural conditions can complicate the investigation and the functions associated with Aβ, Tau, and α-syn under co-morbidity conditions. Artificially induced overexpression of exogenous genes and the manipulation of gene expression may obscure or modify the progression of disease and the interplay among these proteins within tissue interactions, thus hindering the ability to establish straightforward analogies with human LBD pathology.

Despite these challenges, animal models remain an indispensable tool deciphering the pathophysiology of LBD. They furnish a controlled environment in which to examine the implications of genetic mutations and proteinopathies, enabling researchers to delineate the progressive stages of the disease and to evaluate potential therapeutic interventions. The knowledge obtained from these models is invaluable for directing future translational research endeavors aimed at formulating efficacious therapies for LBD patients.

## Summary of co-pathology of Aβ, α-syn, tau, and TDP-43 in LBD models

LBD is characterized by the pathological accumulation of Aβ, α-syn, Tau, and TDP-43 aggregates, which collectively disrupt synaptic function, induce oxidative stress, and impair proteostasis. Oligomeric α-syn propagates through cell-to-cell transmission, seeding aggregation in interconnected brain regions [271]. Furthermore, Aβ oligomers act as nucleation seeds, enhancing α-syn aggregation [220]. This interaction between Aβ and α-syn undermines membrane integrity, exacerbates mitochondrial dysfunction, and activates neuroinflammatory pathways [220]. Additionally, Aβ impairs autophagy-lysosomal degradation, thereby promoting α-syn accumulation [220, 272].

Tau is mainly function in maintaining cytoskeletal network, while co-aggregation of α-syn and Tau generates hybrid oligomers that synergistically disrupt microtubule stability and axonal transport [256]. Similarly, TDP-43 and α-syn synergistically interact to form neurotoxic hybrid fibrils [273], while α-syn function as a Pickering agent in modulating TDP-43 proteinopathies [274]. The overwhelming accumulation of these misfolded proteins impairs the ubiquitin-proteasome system and autophagy-lysosome pathway [275, 276], further activating neuroinflammation, impairing mitochondrial function, and increasing ROS production. These cross-seeding mechanisms facilitate trans-synaptic propagation of pathology [256].

The co-pathology of Aβ, α-syn, Tau, and TDP-43 in LBD represents a complex interplay of cross-seeding, proteostasis collapse, neuroinflammation, and synaptic failure. These interactions create a self-reinforcing cascade that accelerates neurodegeneration. Targeting shared pathways, such as enhancing the functions of ubiquitin-proteasome and autophagy-lysosome, inhibiting kinases, or employing anti-inflammatory strategies, may offer therapeutic potential. Further investigation into the molecular crosstalk between these proteins will refine biomarkers and inform disease-modifying approaches for LBD.

### Clinical treatment

Due to the lack of disease-modifying therapies, LBD management focuses on symptom-based interventions, which require cautions prescribing due to potential significant adverse effects. The most predominantly employed class of medications includes acetylcholinesterase inhibitors, which enhance cognitive function and mitigate cholinergic deficits [277]. Additionally, agents such as memantine have shown efficacy in improving attention and episodic memory [278], whereas levodopa is utilized to reduce parkinsonian symptoms [279], and pimavanserin has been found to alleviate psychosis associated with LBD [280]. A patient-specific approach prioritizing individual tolerance and comorbidities is critical for optimizing therapeutic efficacy and safety.

### Cognitive function

Cholinesterase inhibitors (ChEIs) represent a pivotal therapy for LBD, demonstrating efficacy in mitigating cognitive and neuropsychiatric symptoms while preserving motor function stability [281]. These agents function by elevating brain acetylcholine levels, thereby influencing cognitive processes, functional capabilities, and behavioral outcomes.

Rivastigmine and donepezil are two acetylcholinesterase inhibitors (AChEIs) that inhibit acetylcholinesterase activity and elevate acetylcholine levels. Clinical trial data indicate that rivastigmine (12 mg/day) demonstrates significant cognitive and memory improvement in DLB patients after 20 weeks of administration [282]. Additionally, after 12 weeks of donepezil treatment in DLB patients, the donepezil group showed a fluctuating cognition score improvement in neuropsychiatric inventory test [283]. These medications are generally well-tolerated by LBD patients without exacerbating motor symptoms. However, gastrointestinal side effects (nausea, diarrhea, vomiting, weight loss) are common. While their impact on neuropsychiatric symptoms can be variable, leading to improvement in apathy, cognitive decline, delirium, and visual hallucinations [7].

Memantine, an N-methyl-D-aspartate (NMDA) receptor antagonist that stabilizes glutamate signaling through voltage-dependent, non-competitive inhibition, serves as an alternative option for moderate-to-severe LBD patients and those intolerant to AChEIs [278, 284], while its mechanism of reducing excitotoxicity via attenuated neuronal overactivation that improves cognitive function [285, 286]. Additionally, memantine modulates neuroinflammation, inhibits tau protein hyperphosphorylation to protect neuronal integrity, and promotes neuroplasticity by enhancing brain-derived neurotrophic factor (BDNF) expression and synaptic function, thereby facilitating cognitive recovery [287–289]. Clinical trial data indicate that memantine demonstrates significant efficacy in improving cognitive function in patients with AD. Specifically, 29% of AD patients receiving memantine treatment demonstrated cognitive improvement or no deterioration, compared to 10% in the placebo group [290, 291]. While some researchers also found that memantine potential benefits in enhancing cognitive performance among patients with PDD and DLB, however, the therapeutic effects observed in these patients did not achieve statistical significance when compared to placebo controls [292, 293]. Consequently, there is an urgent clinical need for the development of novel, efficacious, and safe therapeutic approaches specifically tailored for LBD.

### Neuropsychiatric symptoms

Pharmacological management of neuropsychiatric symptoms are limited due to the increased risk of neuroleptic sensitivity observed in LBD patients. Neuroleptic medications are characterized by substantial toxicity and lack sufficient evidence of efficacy within the dementia patient population [14]. Antipsychotics are associated with heightened mortality due to extrapyramidal effects, cognitive decline, falls, stroke, and metabolic disturbances [14].

Cholinesterase inhibitors such as donepezil, rivastigmine, and galantamine are the prior choice for mild to moderate neuropsychiatric symptoms in LBD [294]. A randomized controlled trial demonstrated that 12-week donepezil treatment resulted in improvements of delusions and visual hallucinations [283]. Notably, these findings were further corroborated by a 3-year prospective cohort study, which revealed exceptional clinical efficacy of donepezil against DLB associated visual hallucinations, that achieving almost complete clinical remission rate [295]. Furthermore, rivastigmine demonstrated comparable therapeutic benefits in managing psychotic symptoms: Clinical observations following a 20-week standardized treatment protocol showed that over half of patients achieved near-complete remission in hallucinatory and delusions without exacerbating motor symptoms [282, 296, 297]. Besides, memantine may improve neuropsychiatric outcomes [292], but atypical antipsychotics should be considered when psychotic symptoms escalate to pose serious risks to the patients.

LBD patients exhibit severe hypersensitivity to antipsychotics, which may intensity motor and cognitive symptoms [7]. Quetiapine and clozapine are antagonists of 5-HT_2A_ receptors and dopamine D2 receptors, and this action is proposed as a key mechanism for their antipsychotic effects in schizophrenia without inducing parkinsonism [298–300]. While low-doses of quetiapine and clozapine are sometimes employed to manage psychosis in LBD [301]; clozapine has shown efficacy in PD [302], but still lack of randomized clinical trials for LBD. Most antipsychotics are contraindicated in neuropsychiatric symptoms due to reduced D2 dopamine receptor levels, leading to heightened sensitivity in LBD patients [303]. Pimavanserin, a 5-HT_2A_ inverse agonist, has shown promise in reducing psychotic symptoms in PD and is well-tolerated in LBD. Recent clinical results indicate a significant reduction in the relapse rate of dementia-related psychosis [304], providing an inspiring outcome that offers a promising avenue for the management of psychotic symptoms in LBD.

Antidepressant therapy should be carefully selected in patients with LBD due to the variable risk of adverse effects. While tricyclic antidepressants (TCAs) carry a high risk of anticholinergic side effects, which are more strongly associated with cognitive worsening than exacerbation of motor symptoms [305]. In the management of depressive or anxious states in LBD, selective serotonin reuptake inhibitors (SSRIs) like citalopram, serotonin-norepinephrine reuptake inhibitors (SNRIs) like desvenlafaxine and duloxetine, as well as mirtazapine (monoamine receptor antagonist), are commonly employed, but within a framework of careful consideration and vigilant monitoring [7]. SSRIs and SNRIs prolong the duration of 5-HT and norepinephrine in the central nervous system synaptic cleft by inhibiting their reuptake by presynaptic neurons. This enhances the activation of postsynaptic receptors by these two monoamine neurotransmitters, increases the activity of postsynaptic neurons, and thereby exerts therapeutic effects on depression and other mental disorders [306, 307]. As there are currently no randomized controlled trial data specifically targeting the depressive symptoms of LBD, the selection of medications needs to be adjusted based on the individual patient’s tolerance and response. A personalized approach is advised, prioritizing agents with minimal anticholinergic activity while balancing symptom-specific benefits in LBD treatment.

### Parkinsonism

Motor dysfunction impacts up to 85% of LBD patients [308]. The expression of Parkinsonism in PDD can range from moderate to severe, often necessitating long-term, high-dosage regimens of antiparkinsonian medications, which are accompanied by a considerable risk of adverse effects [309]. Levodopa, the gold-standard treatment for mitigating Parkinsonism in LBD, functions as a dopamine precursor that crosses the blood-brain barrier (BBB) and is converted to dopamine via decarboxylation in the brain; however, it exhibits reduced responsiveness compared to its efficacy in PD [310, 311]. Clinical trial results showed that the effectiveness of levodopa on motor function in DLB was only observed in one-third of the subjects [279, 312]. Moreover, its usage may increase the risk of motor fluctuations and psychosis; however, low-dose regimens with gradual dose adjustments can balance motor symptom relief and psychiatric stability [311]. Prior to initiating levodopa therapy in LBD patients, essential pre-treatment assessments should include screening for bone mineral density and vitamin D deficiency to mitigate fragility fractures risk, alongside implementation of fall-prevention strategies [279]. Moreover, there is an immediate need for the advancement of alternative strategies to address the multifaceted Parkinsonism symptoms in LBD.

### Sleep behavior disorder

LBD patients often experience sleep disturbances which commonly manifest as excessive daytime sleepiness and REM sleep behavior disorder (RBD) [313]. Clonazepam is commonly used for the treatment of both idiopathic and secondary RBD. While in LBD patient with RBD, its use must be approached with caution due to the potential for increased falls and cognitive impairment [314]. Melatonin has demonstrated efficacy in mitigating RBD, and should be prescribed separately to prevent a rise in adverse occurrences specific to LBD patients [315].

Patients with LBD frequently experience hypersomnia, which is exacerbated by nocturnal sleep disruption, sleep apnea, periodic limb movements, related arousals, and dysregulation of intrinsic sleep-wake mechanisms [75]. Modafinil and armodafinil pharmacological agents indicated for the treatment of hypersomnia, involve enhancing dopamine levels in the brain by inhibiting dopamine transporters [316]. Notably, these medications are thought to have minimal interactions with dopamine receptors, making them potential candidates for addressing excessive daytime sleepiness in LBD [317]. However, their efficacy in treating hypersomnia in the context of LBD is still a subject of ongoing research.

### Rehabilitation

Given the absence of disease-modifying treatments for LBD, rehabilitation is recommended as a central strategy for the public health response to dementia [318]. Rehabilitation services are universally acknowledged as a pragmatic framework that can significantly enhance the independence and community engagement of individuals receiving dementia care [319]. Evidence underscores the critical role of physical exercise in dementia care with half-hour session of exercise (three times per week) significantly boost cognitive function, reduce frailty, and lower depressive symptoms in older adults [320, 321].

Cognitive rehabilitation is a customized intervention aimed at improving daily functioning by addressing cognitive deficits [322]. This involves collaboration between therapists, patients, and caregivers to establish practical goals by utilizing evidence-based strategies such as environmental adjustments and compensatory tools to optimize real-life outcomes [322]. Clinical studies have demonstrated the benefits of personalized cognitive rehabilitation in enhancing the daily functioning of those in the early stages of dementia, suggesting that cognitive rehabilitation interventions may hold potential in optimizing cognitive function among LBD patients [323].

Given the prevalence of spontaneous parkinsonian motor features with gait abnormalities in LBD patients, gait training focused on daily living activities, such as sit-to-stand transfers, walking, and turning, is beneficial for mobility enhancement and is preferred over resistance and flexibility exercises [324]. Concurrent cognitive interventions during walking may further optimize gait function [325]. Progression of tasks should be increased as individuals improve to refine gait speed and walking coordination during training processes [325].

Given the inadequacy of pharmacological treatments for neuropsychiatric symptoms in LBD, non-pharmacological interventions, offer viable alternative [318]. Music and massage therapies show efficacy in managing depression and anxiety, while aromatherapy, light therapy, TMS (transcranial magnetic stimulation), and TENS (transcutaneous electrical nerve stimulation) have limited efficacy [326]. Short-term group psychological interventions also alleviate depression and enhance the quality of life in dementia populations [327].

Together, determining molecular targets that are unique to LBD and deepening our understanding of the pathophysiology are of distinct importance for defining current treatment approaches and for the formulation of future therapeutic interventions.

## Future perspectives

Currently, no medications are solely approved for the treatment of LBD, and most therapeutic strategies are derived from the initial clinical trials conducted within the realms of AD and PD. A pivotal challenge in the treatment of LBD lies in the inadequate comprehension of its etiology. Despite a wealth of research suggesting that the interplay between Aβ, Tau, and α-syn contributes to neurodegeneration, the exact modifiers facilitating these protein interactions remain elusive. The underlying mechanisms for the concurrent presence of these proteins in LBD, as well as the influence of genetic risk factors on the emergence of these misfolded proteins, are poorly characterized. Moreover, it remains speculative whether LBD predominantly arises from genetic or sporadic causes. To elucidate the influence of genetic determinants in LBD, it is important to identify families with a prominent genetic susceptibility and to conduct long-term follow-up investigations in future endeavors.

Most of the in vivo research to date has been conducted in mouse models, which have demonstrated the ability to recapitulate certain neuropathological and behavioral abnormalities characteristic of LBD. These models are valuable for distinguishing disease specific molecular differences between AD, PD, and LBD. However, more accessible and convenient models, such as *C. elegans*, *Drosophila*, and *Zebrafish*, should be further developed to expedite the characterization of genetic interactors and the understanding of pathogenesis. Effective models not only enhance pathogenesis exploration but also prove instrumental in drug development. Building upon the existing comprehension of LBD pathogenesis, we generalize several pivotal questions that remain to be fully addressed within ongoing research to provide critical insights into the etiology, progression, and potential treatment modalities for this multifaceted neurological disorder.

Firstly, the identification of biomarkers for LBD is of paramount importance, given that current diagnostic methods are heavily reliant on clinical symptoms. The aggregation of misfolded pathological proteins such as Aβ, Tau, α-syn, and TDP-43 is a universal feature in most LBD cases [18, 328, 329]. However, in vivo detection of these aggregates presents a substantial challenge. Innovative research focuses on the development of specific ligands for PET scans. Aβ PET tracers, such as ^18^F-florbetapir, ^18^F-florbetaben, and ^18^F-flutemetamol, which selectively label Aβ plaques in cortical regions, have gained approval for potential AD detection [330]. Additionally, Flortaucipir (Tauvid™) marks a milestone as the first-approved Tau PET tracer, enabling the quantification and mapping of Tau neurofibrillary tangles in the brains of adult AD patients [331]. Despite this progress, the density of α-syn aggregates in PD brains is generally lower than that of Aβ and Tau in AD brains, complicating the development of specific α-syn PET tracers [332]. Nowadays, no useful α-syn PET tracer has been identified. However, studies had reported the identification of ^18^F-F0502B, a brain-permeable and rapid-washout PET tracer with high affinity for α-syn and preferential binding to α-syn aggregates in brain sections, which effectively imaged α-syn deposits in mouse and non-human primate PD models [333]. More recently, other small-molecule ligands, such as ^18^F-ACI-12,589 and ^18^F-C05-05, had been developed for in vivo visualization of α-syn aggregates [334, 335]. PET imaging with ^18^F-C05-05 had demonstrated high-affinity binding in the midbrains of patients with PD and DLB, representing an innovative imaging technique that holds the potential to significantly advance diagnostic and therapeutic research in PD and LBD [335]. Future holds promise for the emergence of more sensitive, specific, and reliable biomarkers for the detection and monitoring of LBD.

Recent years have witnessed significant advancements in the exploration of α-syn biomarkers in cerebrospinal fluid (CSF) and skin, offering transformative potential for the diagnosis of LBD. CSF α-syn levels have been demonstrated to significantly correlate with disease duration in DLB [336], with studies showing that marked reductions in CSF α-syn protein are detectable even in DLB patients with mild cognitive impairment [337], suggesting its role as an early pathophysiological marker. Other research showed that extracellular α-syn aggregates accumulating in peripheral tissues, such as cutaneous autonomic nerve fibers and sweat glands, which mirror central nervous system in Lewy body pathology [338]. Non-invasive methodologies, including microneedle-based skin sampling and dermal microdialysis, have further expanded peripheral biomarker discovery by enabling minimally invasive extraction of metabolites from skin [339, 340]; therefore, such techniques would be useful to reinforce the potential of skin-derived biomarkers for LBD diagnosis. However, challenges persist in standardizing sampling protocols and validating longitudinal biomarker trajectories across diverse cohorts, necessitating further multicenter studies to establish their clinical utility.

Secondly, treatment strategies based on LBD pathogenesis are imperative. Autophagy, a degradation process targeting damage organelles and misfolded proteins via lysosomes, is perturbed by mutations in genes regulating this pathway, contributing to various neurodegenerative diseases [276, 341]. Our previous studies had demonstrated a pronounced impairment in lysosomal function within the *C. elegans* LBD model, corroborating findings in DLB patients [151]. Mutations in the *GBA* gene, a significant risk factor for LBD, severely compromise lysosomal activity and degradation capacity [195]. Targeted approaches to augment the clearance of misfolded proteins and aggregates are therefore critical to reinforce proteostasis in vivo. The activation of extracellular heat shock proteins (HSPs) had been demonstrated to exert neuroprotective effects by facilitating the disassembly, refolding, sequestration and degradation of misfolded proteins [342–344]. Additionally, the strategic manipulation of chaperone-mediated autophagy to enhance the clearance of misfolded proteins represents a promising therapeutic approach for the treatment of LBD [276]. Furthermore, the exploration of natural products or constituents from traditional Chinese medicine that may positively influence proteostasis should be pursued [345]. Compounds such as fulvic acid and epigallocatechin gallate, derived from natural sources, had shown potential in reducing protein aggregation and may emerge as promising therapeutics for LBD [346]. This research underscores the development of pharmacological agents that modulate autophagy-lysosome function or enhance endogenous clearance pathways as a promising therapeutic strategy for LBD.

Thirdly, the development of novel treatments is essential. One promising approach is the advancement of immunotherapies that may specifically target to misfolded proteins, including Aβ, Tau, α-syn, and TDP-43. This encompasses monoclonal antibodies, peptide vaccines, RNA interference (RNAi) therapeutics, epigenetic interventions, and misfolded protein inhibitors [347, 348]. RNAi has emerged as a promising strategy for the degradation of misfolded proteins, with successful application in the treatment of hereditary transthyretin-mediated amyloidosis [349, 350]. Given this success, the design and evaluation of siRNAs that can specifically target Aβ, Tau, α-syn, or TDP-43 for degradation should be pursued as part of LBD treatment. Moreover, Lecanemab, a humanized IgG1 monoclonal antibody, has gained approval for the treatment of AD, targeting soluble aggregated Aβ across various conformations, including oligomers, protofibrils, and insoluble fibrils [351]. The extrapolation of this therapeutic strategy to LBD, given the overlapping pathology of misfolded protein aggregation and deposition, warrants thorough investigation. Besides, since neuroinflammation is one of the pathogeneses of LBD, some of anti-inflammatory drugs may have potential therapeutic value and thus warrant further investigation. In conclusion, the exploitation of RNAi technology, the advancement of targeted immunotherapies, and the inhibition of neuroinflammation hold significant promise for the treatment of LBD. The optimization of these approaches, tailored to the specific pathological features of LBD, is crucial the advancement of clinical care in this area. Further research is essential to determine the efficacy and safety of these innovative treatment modalities and to translate them into meaningful benefits for patients with LBD.

Furthermore, educational initiatives and public awareness campaigns play a vital role in differentiating LBD from other neurodegenerative diseases, which is crucial for accurate diagnosis and appropriate treatment. Aging represents the predominant risk factor for LBD, and positive anti-aging strategies may potentially mitigate the incidence and progression of the disease. Engaging in routine physical activity, adhering to a balanced diet, and practicing dietary restriction are exemplary lifestyle modifications that could foster a more robust aging process and potentially alleviate the burden of LBD.

## Conclusion

Our review meticulously delineates the distinctions between LBD and other neurodegenerative disorders, elucidating the distinctive pathologies that are hallmarks of the disorder. These include misfolded proteins, Lewy body formation, prion-like propagation, neuroinflammation, dopaminergic and cholinergic degeneration, synaptic transmission impairment, mitochondrial dysfunction, proteostasis impairment, epigenetic changes, unfolded protein response increase, and lipid metabolism aberration. By systematically summarizing findings from animal models of LBD, we reveal that the interactions of Aβ, α-syn, Tau, and TDP-43 function as cross-seeding, inducing proteostasis collapse, neuroinflammation, synaptic failure, and ultimately a self-reinforcing cascade that accelerates neurodegeneration. Furthermore, we highlight the critical challenges in the identifying reliable biomarkers, developing novel therapeutic strategies, implementing educational initiatives, and improving public awareness—all of which are pivotal for advancing LBD treatment. This review not only advances the understanding of LBD pathogenesis but also establishes a robust foundation for current and future research in the field.

## Data Availability

Not applicable.

## Abbreviations

ACh: Acetylcholine
AChEI: Acetylcholinesterase inhibitor
AD: Alzheimer’s disease
A: Amyloid
APP: Amyloid beta precursor protein
ApoE: Apolipoprotein E
α-syn: α-synuclein
BiP: Binding immunoglobulin protein
*C. elegans*: *Caenorhabditis elegans*
ChEIs: Cholinesterase inhibitors
CSF: Cerebrospinal fluid
DAT: Dopamine transporter
DLB: Dementia with Lewy bodies
DMCs: Differentially methylated CpGs
DMPs: Differentially methylated promoters
DMRs: Differentially methylated regions
EEG: Electroencephalography
ER: Endoplasmic reticulum
EWAS: Epigenome-wide association study
FLTD: Frontotemporal lobar degeneration
FTD: Frontotemporal dementia
GRP78: Glucose-regulated protein 78
GWAS: Genome-wide association studies
HO-1: Heme oxygenase 1
LB: Lewy body
LBD: Lewy body dementia
LN: Lewy neurite
MAPT: Microtubule-associated protein tau
mGluR5: Metabotropic glutamate receptor 5
MIBG: ^123^Iodine-metaiodobenzylguanidine
MTBD: Microtubule-binding domain
mtDNA: Mitochondrial DNA
NMDA: N-methyl-D-aspartate
NRF2: Nuclear factor erythroid 2-related factor
NFTs: Neurofibrillary tangles
PD: Parkinson’s disease
PDD: Parkinson’s disease dementia
PET: Positron Emission Tomography
REM: Rapid eye movement
RBD: REM sleep behavior disorder
RNAi: RNA interference
ROS: Reactive oxygen species
SNc: Substantia nigra compacta
SNRI: Serotonin-norepinephrine reuptake inhibitor
SPECT: Single-Photon Emission Computed Tomography
SSRI: Selective serotonin reuptake inhibitor
TCAs: Tricyclic antidepressants
TDP-43: Transactive response DNA-binding protein-43
TENS: Transcutaneous electrical nerve stimulation
TMS: Transcranial magnetic stimulation
UPR: Unfolded protein response
VaD: Vascular Dementia
5-HT: Serotonin
