# Correction: Lewy body dementia: exploring biomarkers and pathogenic interactions of amyloid β, tau, and α-synuclein

**DOI:** 10.1186/s13024-025-00902-4

**Published:** 2025-10-09

**Authors:** Jingfeng Liang, Rongzhen Li, Garry Wong, Xiaobing Huang

**Affiliations:** 1 College of Pharmacy, Shenzhen Technology University, Shenzhen, 518000 China; 2 Department of Neurology, Baiyun District People’s Hospital of Guangzhou, Guangzhou, 510000 China; 3 Department of Global Public Health and Medicinal Administration, Faculty of Health Sciences, University of Macau, Macau S.A.R, 999078 China

**Correction to: Molecular Neurodegeneration (2025) 20:90**

10.1186/s13024-025-00879-0

The original article has been updated to correct the framing of Figs. 1 and 2, as well as to restore the legend of Fig. 2 which was mistakenly incorporated into the main body text.

